# Liver X Receptor as a Possible Drug Target for Blood-Brain Barrier Integrity

**DOI:** 10.34172/apb.2022.050

**Published:** 2021-08-14

**Authors:** Mahsa Eskandari, Ali Awsat Mellati

**Affiliations:** ^1^Medical School, Zanjan University of Medical Sciences, Zanjan, Iran.; ^2^Zanjan Metabolic Diseases Research Center, Zanjan University of Medical Sciences, Zanjan, Iran.

**Keywords:** Blood-brain barrier, Liver X receptor, Drug delivery, LXR, BBB

## Abstract

***Purpose:*** blood-brain barrier (BBB) is made of specialized cells that are responsible for the selective passage of substances directed to the brain. The integrated BBB is essential for precise controlling of the different substances passage as well as protecting the brain from various damages. In this article, we attempted to explain the role of liver X receptor (LXR) in maintaining BBB integrity as a possible drug target.

***Methods:*** In this study, various databases, including PubMed, Google Scholar, and Scopus were searched using the following keywords: blood-brain barrier, BBB, liver X receptor, and LXR until July, 2020. Additionally, contents close to the subject of our study were surveyed.

***Results:*** LXR is a receptor the roles of which in various diseases have been investigated. LXR can affect maintaining BBB by affecting various ways such as ATP-binding cassette transporter A1 (ABCA1), matrix metalloproteinase-9 (MMP9), insulin-like growth factor 1 (IGF1), nuclear factor-kappa B (NF-κB) signaling, mitogen-activated protein kinase (MAPK), tight junction molecules, both signal transducer and activator of transcription 1 (STAT1), Wnt/β-catenin Signaling, transforming growth factor beta (TGF-β) signaling, and expressions of Smad 2/3 and Snail.

***Conclusion:*** LXR could possibly be used either as a target for drug delivery to brain tissue or as a target for maintaining the BBB integrity in different diseases; thereby the drug will be conducted to tissues, other than the brain. If it is verified that only LXRα is necessary for protecting BBB, some specific LXRα ligands must be found and then used in medication.

## Introduction

The blood-brain barrier (BBB) is a pivotal cellular structure necessary for the preservation of neuronal homeostasis. Moreover, the BBB is an extremely selective border that allows the transition of specific molecules via the passive diffusion and different nutrients such as amino acids, glucose, electrolytes, and vitamins, which are essential for neurons’ activity.^1^ Dense contacts among endothelial cells (capillary wall, pericyte, and end feet of astrocyte that is a capillary scabbard), and tight junctions consequently form the BBB. Efflux transporters, especially ABC transporters, could supplement the BBB.^2,3^ In this regard, it was indicated that ABC transporters pump out lots of foreign compounds like pharmaceutical materials from the brain tissue.^4^ ABC transporters contribute into the manifestation of multidrug resistance (MDR). In MDR, patients are resistance against both drugs that are currently taking as well as the different types of drugs.^5^ It was approximated that almost all large molecules and more than 98% of small molecules’ medicine are unable to bypass BBB.^6^

Given this fact, neurons are electrically stimulated cells requiring precise electrophysiological and chemical controls to have a proper function. Therefore, the brain needs a balanced, regular, and precise microenvironment.^7^ Therefore, the BBB forbids some potentially harmful causes such as immune cells, pathogens, and toxins to enter the brain tissue from the blood.^8^

BBB dysfunction is involved in various neurological diseases (including brain tumors, stroke, multiple sclerosis, epilepsy, Alzheimer’s disease (AD), inflammation, and infection), which occurs at late and early stages of any disease’s progression. There are two specific types of BBB dysfunction as follows: 1- Increased permeability accompanied by edema. 2- Extensive cellular permeability throughout the BBB.^9^

Based on some previously performed studies, liver X receptor (LXR) is necessary for stabilizing the integrated BBB in various ways. It was shown that LXR activity could lower BBB permeability and brain edema.^10^ Moreover, LXR is related to various signaling such as nuclear factor-kappa B (NF-κB), mitogen-activated protein kinase (MAPK), transforming growth factor beta (TGF-β), and Wnt/β-catenin signaling.

In this study, we aimed to express the role of LXR in maintaining the integrated BBB as well as in its related mechanisms. It was indicated that LXR can be targeted either for drug delivery to the brain tissue or for maintaining the BBB in different diseases to transfer the drug to an organ other than the brain. Albeit, in the current study, we found a study showing that LXR agonists do not influence paracellular permeability properties.^11^

## LXR activity

These are among the transcription factors activated by ligands and pertain to nuclear receptor superfamilies. LXR contributes in the processes of immune responses and cell’s metabolism, differentiation, and proliferation.^12^

The LXR acts in combination with a Retinoid X receptor (RXR). Accordingly, this heterodimer attach to LXR response elements (LXREs) in LXR target genes. Thereafter, LXRE comprises the sequences (AGGTCA) on repeat that can be distinguished by four nucleotides (DR4). Of note, RXR or LXR agonists could activate this heterodimer.^13^ Simultaneous utilization of both RXR and LXR agonists causes a more intense response compared to when using each agonist alone.^14^ After ligand binding, the co-repressors (silencing mediator used for retinoic acid, thyroid hormone receptor, and nuclear receptor co-repressor) are released. Thereafter, co-activators are recruited to bind to the complex, which then leads to gene transcription.^15^ LXR is activated by endogenous (cholesterol derivatives) or synthetics (GW3965 and T0901317) agonist. Correspondingly, synthetics agonists stimulate LXR stronger.^10^ in addition, RXR is activated by 9-cis-13, 14-dihydroretinoic acid derived from vitamin A.^16^ Another ligand of RXR is Docosahexaenoic acid (DHA) detected in the brain of mammalian.^17^

## LXR isoforms

LXR has two isoforms called LXRβ and LXRα that have a great similarity with each other.^18^

LXRα is synthesized by some tissues such as the kidney, intestine, adrenal gland, adipose tissue, lung, macrophages, spleen, and BBB, which is mostly expressed by the liver while LXRβ is expressed by almost all tissues. Akanuma et al announced that mRNAs of two LXR isoforms are synthesized by endothelial cell line of the immortalized brain capillary.^19,20^ Notably, the human LXRα and LXRβ genes are placed on 11p11.2 and 19q13.33 situations, respectively. (https://www.ncbi.nlm.nih.gov/gene)

A previous study found that LXRα is present in lower levels in the mice endothelial cells and the hCMEC/D3 cell line, compared to LXRβ, and LXRαis necessary for protecting BBB not LXRβ.^21^

In mouse models, LXR-623, LXRα-partial/ LXRβ-full agonists via LXRβactivation induced expression of the ATP-binding cassette transporter A1 (ABCA1) as well as the inducible degrader of the LDLR (IDOL), which respectively led to cholesterol efflux and prevented cholesterol up-taking through both LDLR ubiquitinating and degradation. IDOL modulates the levels of both the VLDL receptor and the Apo E receptor 2. Moreover, it is expressed in neurons and it was reported that it can inhibit neurite outgrowth. Presumably, LXR-623 kills glioblastoma cells by reducing cellular cholesterol. As well, LXR-623 was found to have the ability of infiltrating the BBB.^22^

A previous study reported that in rat brains, LXRβ expression level has not significantly changed after exposure to ischemia, whereas the expression of LXRα was very low, which was in line with some reports showing that LXRβ is the predominantly expressed form in brain tissue.^23-25^

Another study on variation between two isoforms is required to confirm or deny these data and paying more attention to this deference in medication.

Most of the ligands used could activate both LXR isoforms. If the difference between the two isoforms is confirmed, specific ligands must be found.

## LXR and ABCA1

One possible mechanism that may be involved in maintaining the integrated BBB is the enhanced ABCA1 expression. In this regard, ABCA1 is one of the LXR target genes. In this study, T0901317 (a LXR ligand) heightened the mRNA of ABCA1 l in TR-BBB13 cells; however, the expression of ABCG2 did not alter.^19^ ABCA1 is a transmembrane protein and a mediator of cholesterol efflux of intracellular. Moreover, it has neuroprotective and anti-inflammatory effects on both brains and the peripheral circulation.^26,27^ In mice model of cerebral artery occlusion, ABCA1 failure leads to the reduced BBB integrity, the injured white matter/axon, and the developed function deficiency. Besides, insulin-like growth factor 1 (IGF1) and aquaporin-4 (AQP4) decreased, while matrix metalloproteinase-9 (MMP9) heightened after stroke in brain ABCA1 deficiency mice.^28^

MMP9 is an important factor in BBB leakage and white matter harm after stroke, which can destroy the extracellular matrix and tight junction.^29^

IGF1 is mostly produced in situ from astrocytes and microglia, because it cannot pass the BBB.^30^ Of note, IGF1 overexpression keeps hippocampal neurons from harm.^31^ Moreover, IGF1 medication into the cerebral lateral ventricle could decrease the BBB leakage and cellular/tissue injuries.^32^

Aquaporin-4 is highly expressed in end feet of astrocyte and then mediates water conduct through the cell membrane as well as protecting the brain against edema.^33^

As stated earlier, ABCA1 facilitates the cholesterol efflux from the cell (macrophages or peripheral tissues) and then delivers cholesterol to apolipoprotein­ A1, pre-β HDL, and apolipoprotein E (apoE). Finally, high-density lipoprotein (HDL) leads to a reverse cholesterol transmission from tissues to the liver, in order to eliminate cholesterol.^34^

HDL is formed in both the bloodstream and brain. As well, HDL has many functions, including anti-inflammatory, anti-oxidant, anti-thrombotic, and modulating immune functions. Besides, the elevated plasma HDL could protect human body against neurodegenerative diseases associated with lipid.^35^ Furthermore, LXR agonists can be effective on preventing AD. For example, it has been shown that performing a treatment with 24*(S)*-hydroxycholesterol (24OH-C), 27OH-C, TO901317, and cholesterol *in vitro* model of the BBB consequently leads to the reduced amyloid- β (Aβ) possibly through increasing expression of LXR target genes.^36,37^ Cholesterol pumped out from cells could be promoted by the ApoA-I/ABCA1 pathway that has been shown to be associated with amyloid-β (Aβ) peptide generation. The deletion of ABCA1 in AD mouse models significantly decreased, ApoE which was related to Aβ precipitations in the brain.^38^ A previous study also reported that, in their cultured neuroblastoma cells, 24S-OHC showed a protective effect on beta-amyloid production, whereas 27OHC appeared to have the opposite effect.^39^ Aβ peptide accumulation in the brain parenchyma and the cerebrovasculature is considered as one of the important hallmarks of AD.^40^

## LXR and cholesterol dysregulation

Because cholesterol cannot cross the BBB, the whole of brain’s cholesterol is approximately made locally via de novo synthesis. Cholesterol is supplied by lipoproteins up-taking and/or by de novo biosynthesis in the peripheral nervous.^41,42^ it is noteworthy that the brain possesses nearly 20% of body cholesterol.^43^ CYP46A1 alters the excessive amount of cholesterol to 24(S)-hydroxycholesterol (24OH-C). Thereafter, 24OH-C passes through BBB and then enters the liver to become bile acids and be eliminated from the body.^44^ The brain rarely generates 27-hydroxycholesterol (27OH-C). So, this mainly enters the brain, and is then developed by CYP27A1.^45^ Both of them (24OH-C and 27OH-C) are powerful stimulators of LXR.^46^ 24S-OHC was removed from rat brain via organic anion transporting polypeptide 2 (oatp2) across the BBB.^47^

LXRs are the key regulators of cholesterol and HDL metabolism involved inthe reverse cholesterol transport in a variety of ways. Of note, LXR has several target genes, including ABCG1, ABCA1(Involved in cholesterol efflux), ADP-ribosylation factor-like 7 (simplifies cholesterol transport to the cell membrane for pumping-out), CYP7A1(Involved in Cholesterol conversion to bile acid), ABCG5, ABCG8 (Involved in the apical efflux of cholesterol from enterocyte that promotes biliary excretion of sterols), APOE (enhances the HDL returning to the liver), and phospholipid transfer protein (PLTP) (exchanges phospholipids from triglyceride-rich lipoproteins with HDL and HDL size regulation).^48^ LXR activity consequently enhances pumping-out of cell cholesterol and HDL-like particles creation. In this research, LXR activation using 24(S)-hydroxycholesterol or TO901317 increased 2.5 fold expression of PLTP as well as its main function. PLTP contributes to HDL genesis and remodeling in BBB.^49^

In BBB model, it was found that LXR agonists (27OHC, (24(S)-OHC, and TO901317) can express ABCG1 and also regulate apoM.^50^ 5% of HDL particles comprise apoM,^51^ which is mostly expressed by liver and kidneys, and then secreted into plasma or fused to lipoproteins.^52^ Overexpression of apoM helps in evaluating plasma HDL cholesterol, preß-HDL formation, cholesterol efflux, and promotes the antioxidant activity of HDL.^53,54^

In aprimary porcine BCEC, LXR ligands reduced the biosynthesis of cholesterol between 30 and 80%, while increased pumping-out of cell cholesterol 2.5 fold via affecting hydroxymethylglutaryl-CoA reductase (HMG-CR), which is a key regulator of cholesterol biosynthesis and ABCA1.^36^ In the above-mentioned model, scavenger receptor class B type 1 was expressed. Accordingly, this is a receptor that uptakes lipids related to HDL. 24-OHC increased expression of this receptor up to almost 1.5-fold. 27-OHC reduced cholesterol levels via decreasing the expression HMG-CR, sterol-regulated element-binding protein-1, and LDL-receptor. As well, up-regulation of the peroxisome proliferator-activated receptors γ (PPAR-γ), LXR-α, ABCA1, and ApoE genes, was observed in C6 glioma cells.^55^ as well, one of the PPAR-γ)’s (as a nuclear receptor) target gene is LXR-α.^56^

In mouse brain endothelioma cell line, pharmacological concentrations of both 9-cis retinoic acid (9cRA) and all-trans retinoic acid (ATRA) (5μM) via LXR-RXR complex consequently up regulated ABCG1 and ApoE.^57^

## LXR and hsp70

Heat shock proteins (HSPs) contributed into protein folding, homeostasis, and survival of the cell.^58^

Additionally, recombinant HSP70 led to cholesterol removal from primary human macrophage. Thereafter, HSP70 bound to the macrophage LXR alpha promoter, which then increased LXR alpha and its target mRNAs, including both ABCA1 and ABCG1. Interestingly, in zebrafish, upon high cholesterol diet, the administration of rHSP70 to the swimming water was not consistent with the above-mentioned results. However, endogenous HSP70 increasing by 17-dimethylaminoethylamino-17-demethoxy-geldanamycin (17-DMAG) promoted cholesterol removal. Moreover, 17-DMAG increased both endogenous HSP70 and ABCA1 proteins’ levels in primary human foam cells.^59^

## LXR and EMT

Another possible mechanism involved in maintaining the integrity of the BBB is epithelial to mesenchymal transition (EMT).^21^ When EMT occurs, modifying the expression of genes and adhesion of cell to each other and cytoskeleton might result in a more migratory and invasive abilities, which could lead epithelial cell to become mesenchymal cell.^60^ In various cancers cell lines, the LXRα overexpression or existence is directly involved in their invasive property.^61,62^

In LXRα mutant mice, TGF-β signaling and intensive expression of Snail protein and Smad 2/3 have been promoted in epithelial cells of ventral prostate. Additionally, in nodules, E-cadherin expression has decreased or lost and epithelial cells underwent EMT. Snail is known as an intensive suppressor of E-cadherin transcription and it also is a marker for EMT.^62^

In some patients with lung cancer, it was observed that the resistance to EGFR inhibitors (such as Gefitinib that is a cancer drug) can be related to the EMT phenomenon.^63^ In non-small cell lung cancer cells, the combined medication of Gefitinib and GW3965 mostly leads to tumor attributes changing, as well as re-sensitizing this cells to the Gefitinib. In addition, this combined medication has reduced IC50 for Gefitinib. Correspondingly, Gefitinib medication increases vimentin expression, while the combination medication was found to result in the reduced vimentin.^64^ Accordingly, it is notable that Vimentin is a medium filament. Vimentin, microtubules, and actin filaments are known as the components of the cytoskeleton. Moreover, Vimentin contributes into cell adhesion, signal transduction, apoptosis, and migration.^65^

In a study conducted on 93 men and 31 women, it was reported that LXRα expression in poorly and undifferentiated gastric cancer tissue is significantly lower than that of well and intermediate differentiated gastric cancer tissue. Tumor cell differentiation was also found to be related to EMT and drug resistance. LXRα presumably promotes the differentiation of human gastric cancer cells via the inhibition of Wnt/β-catenin signaling by reducing the β-catenin expression as well as reducing downstream targets of this pathway, including CD44 and cyclin D1. In this study, same results were achieved from an animal experiment.^66^

## LXR and neuroInflammation

The proinflammatory cytokines and chemokines are pivotal components in the neuroinflammatory process. In the central nervous system, glial cell or leukocyte of these inflammatory agents generate in situation that they are recruited after BBB breakdown.^67^

LXR activation leads to anti-inflammatory actions via the Inhibition of Nuclear factor-κB signaling.^23^ in this regard, NF-κB is a transcription factor mediating the expression of its target genes. NF-κB plays a pivotal role in proinflammatory gene induction.^68^ Moreover, LXR activation in LPS-stimulated astrocytes of mice prohibits the generation of proinflammatory cytokines such as monocyte chemoattractant protein-1(MCP1), IL-6, and IL-1β via the NF-κB pathway.^69^ in addition, MCP1 recruits dendritic, monocyte, and memory T cells in the inflammation location.^70^ As well, it was indicated that LXR agonist could reduce microglia activity (microglia acts as macrophages in the brain) and macrophage inflammatory protein 2 (MIP-2), called CXCL2.^71^ Macrophages and neutrophil exude MIP-2, which is a chemotaxis factor for hematopoietic and leukocyte cells.^72^

**Figure 1:**
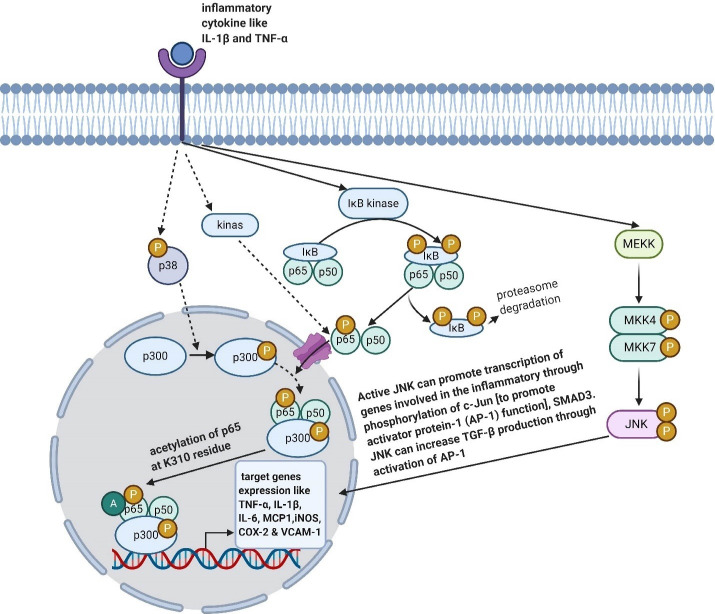
Effects of LXR on some inflammatory pathways. Created with BioRender.com

Decontrolling of NF-κB activity leads to aberrant T-cell activity related to the formation of both the autoimmune and inflammatory conditions. Moreover, NF-κB regulates the differentiation of T-cell as well as effector T cell’s developing and activity. Notably, Th17 and Th1 are inflammatory cells, which are related to autoimmune and inflammatory responses.^73^ In the autoimmune encephalitis mice, the LXR activation via T0901317 (an agonist of LXRα & β, dominant LXRα stimulator) inhibited both T-cell’s proliferation and activation. Additionally, it was reported that IFN-γ mRNA and IFN-γ-dependent MHC class II expressions by microglias have reduced. In ligand treated mice, mRNAs of CXCL-1, MMP-9, TNF-a, and ICAM-1 have also decreased.^74^ After CXCL-1 binding to its receptor, it was observed to activate phosphatidylinositol-4,5-bisphosphate 3-kinase-γ (PI3Kγ)/Akt, MAP kinases such as ERK1/ERK2 or phospholipase-β (PLCβ) signaling pathways.^75^ Interestingly, in the cultured microglia, T0901317 decreased the activities of JNK, p38, MAPK, and NF-κB signaling.^71^ Correspondingly, P38 MAPK and c-Jun N-terminal kinase (JNK) are well-known in MAPK signaling,^76^ The MAPK signaling is started when a stimuli like pro-inflammatory cytokines, starts binding to their receptor on the cell, which then activates p38 and regulates transcriptional activity of NF-κB. Indeed, during neuro-inflammation process of phosphorylated p38, co-activator p300 is activated. Thus, the activated p300 binds to NF-κB and acetylates the p65. As a result, NF-κB-p300 transcriptional complex gains the optimal transcriptional ability.^77^ Eventually, the JNK activation enhances expressions of TNF-α, IL-1β, and IL-6 in microglias^78,79^ (Figure 1).

In interferon gamma stimulated astrocytes, LXR agonists could prevent STAT1-mediated inflammatory responses. SUMOylated LXRs bind to STAT1; therefore, STAT1 could not attach to the promoter sequence of its target genes and expressions of interferon regulatory factor 1, TNF-alpha, and IL-6 would be inhibited.^80^ In macrophage, co-repressor is stabilized by SUMOylated LXR on NF-κB, which reduces the expression of its target genes.^81^

In the Cheng’s study, using rat model, GW3965 (LXR agonist) was found to have a neuroprotection effect, which is related to a meaningful decrease in nuclear move of p65 subunit of NF-kB (happened immediately (12 h) after ischemia). As well, GW3965 medication reduced cyclooxygenase 2 (COX-2) expressions in the hippocampus.^82^ Another study with TO901317 administration to experimental intracerebral hemorrhage mice model also achieved the same results.^71^

The COX-2 could form prostanoids such as thromboxane and prostaglandins from arachidonic acid.^83^ After the occurrence of global cerebral ischemia, prostaglandin E2 causes histopathological modifications in the hippocampus.^84^

Its promoter sequence includes functional tandem NF-κB sites. Notably, vascular cell adhesion molecule-1 (VCAM-1) is an inflammatory cytokine guiding the rolling and inducing the adhesion of leukocytes to vascular endothelium, which consequently leads to diapedesis of leukocytes.^85^ VCAM-1 is highly overexpressed in the inflamed brain. In hCMEC/D3, which is a human cell line, as well as in mice model of MS, specific knockout of LXRα promotes the expression of VCAM-1 that enhances monocytes diapedesis across BBB. Correspondingly, Leukocyte extravasation is considered to be very important in inflammation processes and BBB dysfunction.^21^ T0901317 incubation in peripheral endothelial cell, declined the I-CAM, V-CAM, and E-selection expressions, which are known as adhesion molecules.^86^ VCAM-1 interacts with ezrin (that connects cell membrane to actin involved in adherence and EMT) and moesin. As well, it is an integrin α4β1 agonist.^87-89^

It was indicated that 22(R)-hydroxycholesterol and 7-ketocholesterol via suppressing phosphorylated signal transducer and activator of transcription1/3, interferon regulatory factor-1, and interferon-b, could suppress inducible nitric oxide synthase (iNOS) expression as well as suppressing nitric oxide release.^90^ INOS is recognized as a target gene for NF-κB.^91^ In a rat model, it was shown that iNOS possibly is important in BBB breakdown, cerebral edema, and cell injury. In this regard, iNOS may be involved in the pathophysiology of many central nervous systemdiseases.

In iNOS−/− mice, MHC-II expression significantly decreased on the dendritic cells, but not on macrophages. Dendritic cell and macrophage play an antigen-presenting cell role. Albeit, brain iNOS might be needed for having a proper repair after damage.^92^

According to some previous studies, neuroinflammatory may be involved in CNS damage of Heat stroke.^93^ Microglia acts as a macrophage in the brain. Indeed, these must be able to recognize and swallow foreign bodies.^94^ Moreover, Heat stroke can be activated by NO, ROS, TNF-α, IL-1β, and IL-6 as well as high ambient temperature.^95-98^ In heat stroke animals, the activated microglias have been identified in the brain. MiRNAs are epigenetic gene expression regulators that are used post-transcriptionally. MiR-155 elevates p65 and IκBα phosphorylation, nuclear p65 and also promotes NF-κB activity in the microglias. Thereafter, the prohibition of miR-155 leads to the significant augmentation of mRNA and protein of LXRα as well as the reduced pro-inflammatory cytokines.^99^

As mentioned earlier, ABCA1 is one of the target genes of LXR. In the brain of ABCA1–deficient mice, IGF1 was observed to decrease. Accordingly, IGF-1 has a neuroprotective property, and it can reduce the BBB permeability and suppress immune via programmed cell death protein 1 (*PDCD1*), which is a receptor suppressing immunity. As well, an after-stroke Intra-cerebroventricular injection of IGF-1 to female rats suppressed 2 to 5-fold pro and anti-inflammatory cytokines such as the pleiotropic cytokine, IL-6, IL-13, and CCL2. In this regard, it may have a biphasic function.^32^

## LXR and junctions

Another possible way contributing to BBB integrity maintenance is the up-regulation of tight junction molecules. In the Wouters and colleagues’ study, LXRα knockout resulted in the reduced claudin-5.^21^ Additionally, LXR activation prevented the down-regulation of zona occludens-1 and occludin in ischemic vessels of mice.^10^ in this regard, occludin is an integral membrane protein, which is considered to be important in the stability of tight junction assembly and barrier function.^100^ Moreover, occludin has C-terminus that is critical in receiving and transmitting cell survival signals, as well as in correcting the assembly of tight junction.^101,102^ In addition, the N-terminus of occludin is involved in tight junction sealing/barrier properties.^102^

ZO-1 acts as a scaffold by binding the tight junction (TJ) strand to the actin cytoskeleton. Correspondingly, ZO-1 is placed on a cytoplasmic membrane surface of intercellular tight junctions. This may possibly contribute to signal transduction at cell-cell junctions.^103^

Claudins and occludin are known as major tight junction proteins.^104^ Claudin-5 is dominant in BBB.^105^ The BBB of Claudin-5 knockout mice is damaged and permeable; therefore, the whole deletion of claudin-5 is fatal.^106^

Pharmacological concentrations of both ATRA (5μM) and 9cRA via LXR/RXR signaling stimulated expressions of ZO-1 and VE-cadherin in mouse brain endothelioma cell line. Notably, high ATRA concentrations possibly act through 9cRA.^57^

The integrity of intercellular junctions is known as a major determinant of endothelial permeability, and VE-cadherin-based bonds are of particular importance. Of note, VE-cadherin is a calcium-dependent cell-cell adhesion glycoprotein.^107^

IGF-1 may maintain BBB integrity via increasing electrical resistance via regulating both claudin-3 and occludin in submandibular gland cells,^108^ claudin-1 in osteoblast cell through the MAP-kinase signaling,^109^ and ZO-1 in A431 cell.^110^

## Calpastatin and LXR

Another mechanism involved in the declined brain edema and the reduced BBB leakage is the up-regulation of calpastatin by LXR.^10^ In this regard, calpastatin is known as the important regulator of calpain and a natural calpain-specific inhibitor by having no inhibitory effect on other proteases. Accordingly, calpastatin molecule can be formed from four inhibitor units, each one of which could inhibit one calpain molecule with different efficiencies.^111^ In cerebral arteries obstruction mouse, calpastatin inhibits calpain-1/2, which consequently leads to sustaining of p120 catenin. Calpain-1/2 is a calcium-dependent cysteine protease, which decreases p120 catenin. Thereafter, P120 catenin inhibits*RhoA* and over-activates Cdc42. Moreover, *P120* catenin could also inhibit *RhoA*–GDP dissociation and promote the Cdc42 approach to its guanine exchange factors.^10^ RhoA deactivation decreases stress fiber developing, thereby stabilizing tight junction network.^112^ As well, Cdc42 activity regulates polarity of cells and enhances BBB integration by assembling tightly junctions such as ZO-1 and occluding.^113^

## LXR and medication

LXR activity could elevate the frequency of the ABCB1 and ABCC1 on capillaries of ischemic brain. Recently, it has been shown that the ApoE could regulate the equilibrium between the abluminal ABCC1 and luminal ABCB1 through its receptor, called ApoER2.^114^ Both ABCB1 and ABCC1 are ATP-dependent efflux pumps excreting some xenobiotics and pharmaceutical drugs. So, this MDR should be paid more attention.^3,4^ Considering the above-mentioned ABCB1 and ABCC1 functions, the possibility of the usefulness of LXRα specific ligand as well as the important role of LXR in BBB maintenance can be helpful in proper drug developing according to the remedial goal.

For example, the lack of fully effective treatment options leads to a poor prognosis of glioblastoma.

Therefore, it is very important to obtain enough therapeutic agents to the site of the brain tumor. However, due to the presence of BBB, the way of delivering therapeutic agents to the tumor site is very questionable.

It seems that the local administration of the drug and targeted drug delivery to the brain tissue might also be considered due to the side effects of the LXR agonist on other body tissue such as increasing plasma and liver lipids level.^115^

## Conclusion

The BBB is essential for maintaining brain homeostasis and neural correct activity. BBB dysfunction could significantly involve the pathogenesis of both neurodegenerative and neuroinflammatory diseases. According to recently performed studies, it seems that LXR plays a critical role in protecting BBB against injuries. LXR can also be effective on maintaining BBB by affecting different signaling pathways such as NF-κB, MAPK, TGF-β, and Wnt/β-catenin, leading to the prevention of the proinflammatory cytokines and chemokine production, the inhibition of the EMT and migratory phenotype developing, and maintaining and stabilizing the tight junction molecular network

During the process of neuroinflammation, proinflammatory cytokines and chemokines play very important roles via being produced locally by glial cells or by the recruited leukocytes.

Given that many drug molecules cannot cross the BBB, LXR can be targeted either for drug delivery to the brain tissue or for maintaining the BBB integrity in different diseases, in order to transfer the drug to an organ, other than the brain. It seems that the local administration of the drug and targeted drug delivery to the brain tissue might also be considered due to the side effects of the LXR agonist on other tissue like increasing plasma and liver lipids level. If it is verified that only LXRα is necessary for protecting BBB, some specific LXRα ligands must be found and then used in medication.

## Ethical Issues

Not applicable.

## Conflict of Interest

Authors declared no conflict of interest.
